# Supplementary material for: Structural basis of oncogenic histone H3K27M inhibition of human polycomb repressive complex 2
Source: Nat Commun. 2016 Apr 28;7:11316. doi: 10.1038/ncomms11316 (PMC4853476; doi:10.1038/ncomms11316)
Supplement: Supplementary Information — Supplementary Figures 1-6 and Supplementary Table 1. [file ncomms11316-s1.pdf]

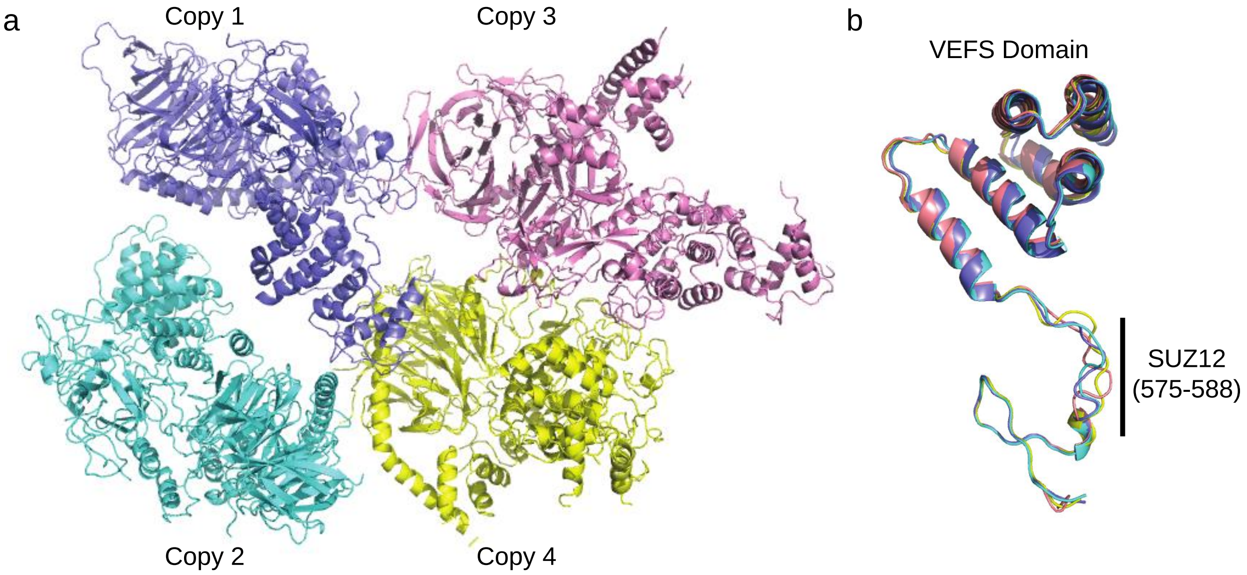

Supplementary Figure 1 – The crystallographic asymmetric unit. Human PRC2 crystallised with 4 copies of the complex in the asymmetric unit (**a**). There was strong electron density of the Jarid2 peptide and for SAH in all four copies. Two of the four NCS molecules contained strong density for the H3K27M peptide, the remaining two molecules showed weaker density suggesting lower occupancy of the peptide in the SET domain in these two copies. The four copies are essentially otherwise equivalent (**b**) The majority of the SUZ12 residues that form the Vefs domains in the four copies in the asymmetric unit align closely, with some differences occurring in the helices adjacent to the Sant2 domain (residues 630-645 and 672-683). Also, it is clear that the SUZ12 residues 575-588, which form an extended strand that interacts with the SAL, show much more variability in their position. It is particularly interesting to note that three residues (E584, E586, and D588) previously shown to mediate histone H3 (31-42) binding[13] and stimulation of PRC2 lie in this more variable region.

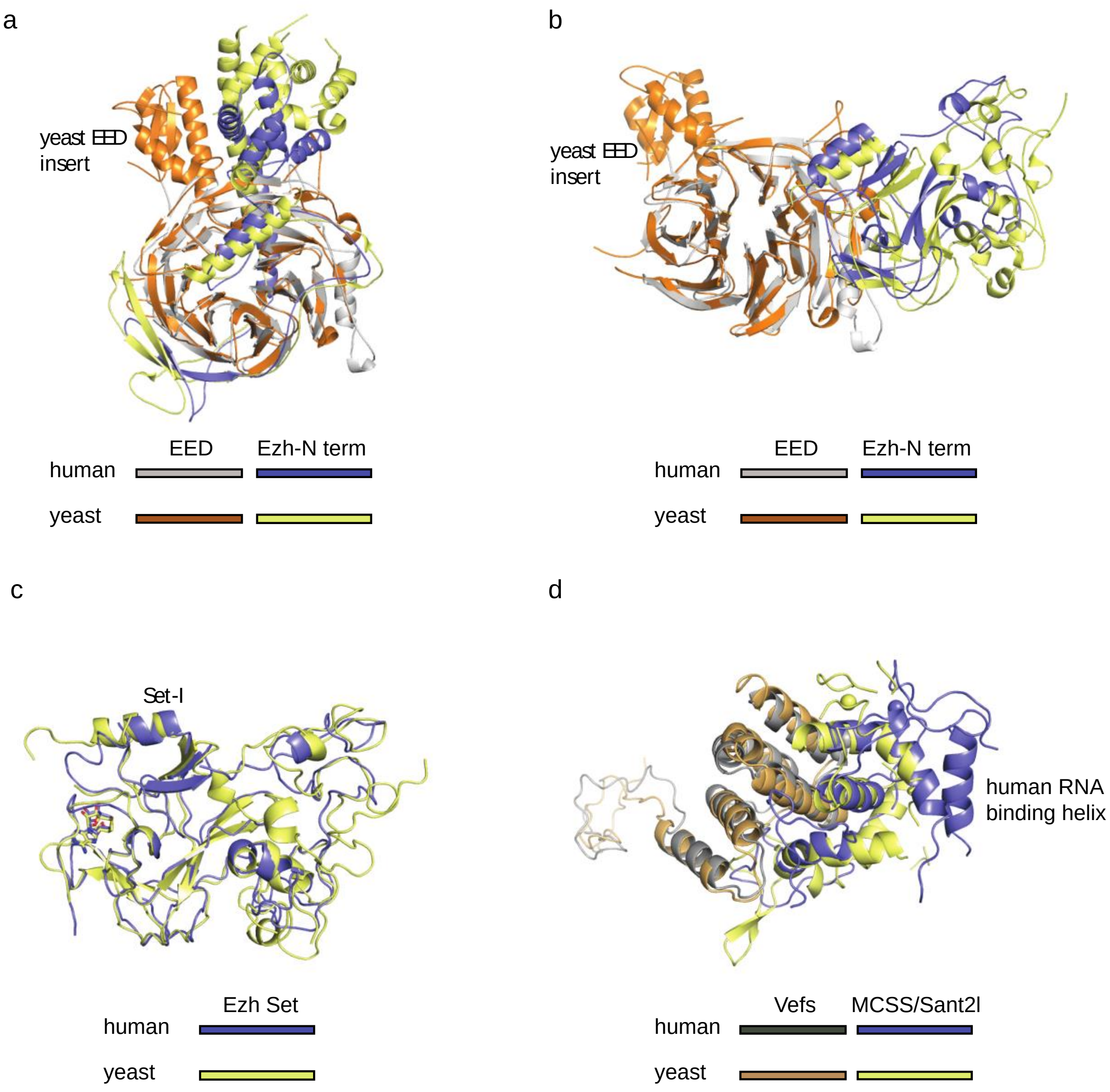

Supplementary Figure 2 - Comparison of the overall structures of human and *Chaetomium thermophilum* PRC2. Overall there is good structural conservation between the human and yeast PRC2. However alignments of the structures on individual domains identify rigid domain movements. The structures are represented in cartoon format and the colour schemes for the individual figures are shown in the accompanying key. (**a**) Comparison of the human and yeast N-terminal regions of Ezh, (including SBD, EBD, BAM, SAL, SRM and SANT1 regions), after alignment on EED molecules. The yeast EED has an extra insertion domain, which is absent in human. (**b**) Comparison of human and yeast CXXC and SET domains after alignment on EED. (**c**) Human and yeast CXXC and SET domains aligned together. (**d**) Comparison of human and yeast middle lobes after alignment on SUZ12 Vefs domain, the MCSS and SANT2 regions are shown. Human PRC2 contains an RNA binding helix that is absent in yeast.

a

b

c

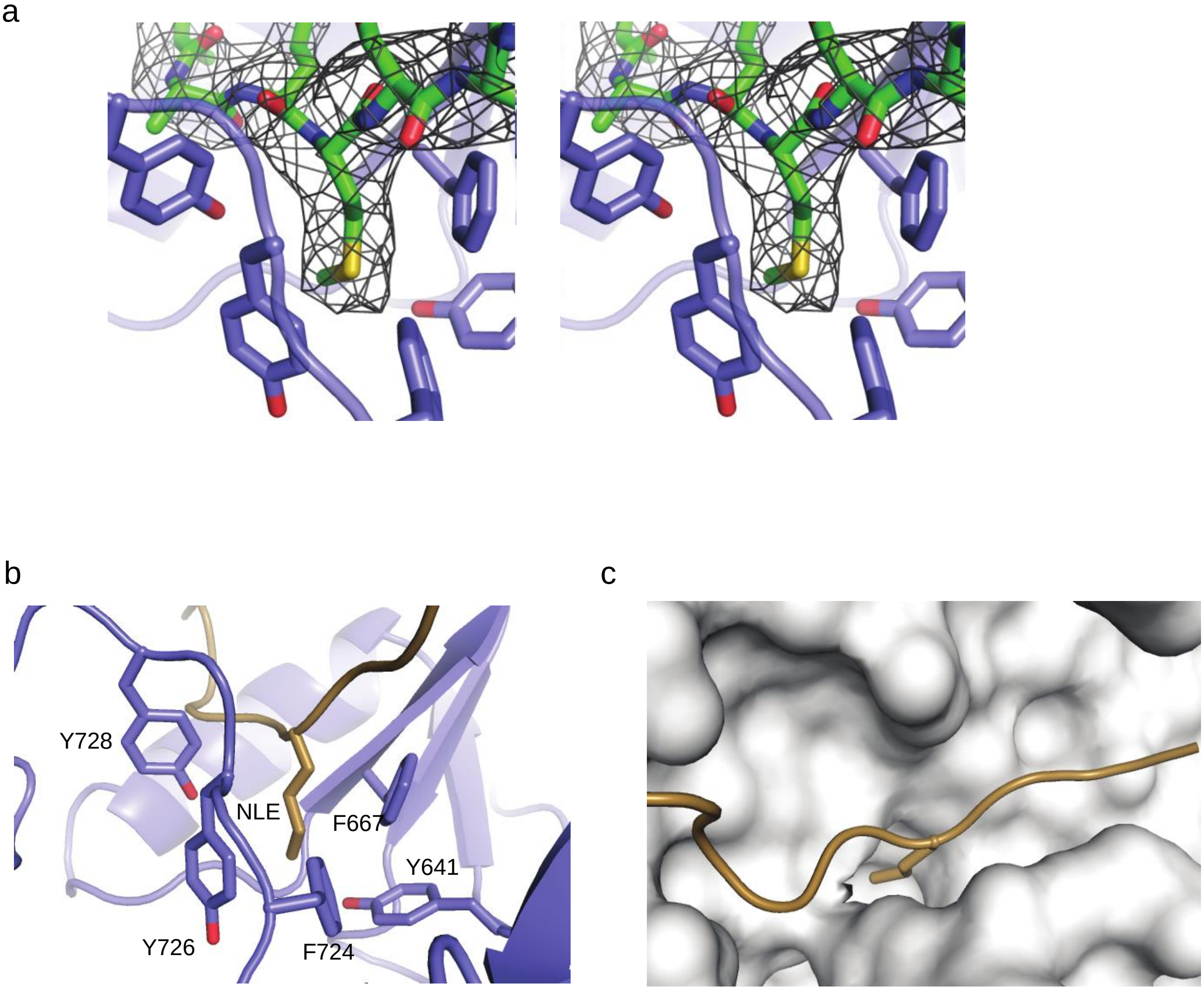

Supplementary Figure 3 – The lysine binding channel favours hydrophobic side chains. **(a)** Stereo representation of the EZH2 lysine binding channel showing the omit density for the peptide contoured at 2σ. (**b**) (**c**) The spatial and chemical characteristics of the “lysine” binding channel favours unbranched side chains of a hydrophobic character. This reflects the requirement of a deprotonated Nε substrate for the methyl transfer reaction. It also explains why residues such as methionine or norleucine at position 27 exhibit strong inhibition in kinetic studies[19].

a. Direct Titration of labelled peptide

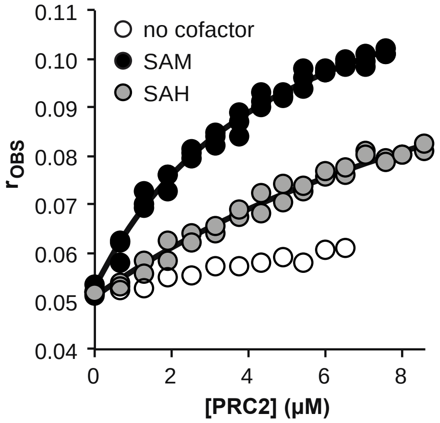

Peptide	Additive	Kd (μM)	rL	rPL
FAM-K27M	320 μM SAM	4.3 (0.4)	0.053 (0.001)	0.129 (0.002)
FAM-K27M	1.3 mM SAH	14.6 (3.8)	0.051 (0.002)	0.136 (0.003)
FAM-K27M	none	> 50 μM	ND	ND

b. Titration with unlabelled peptides

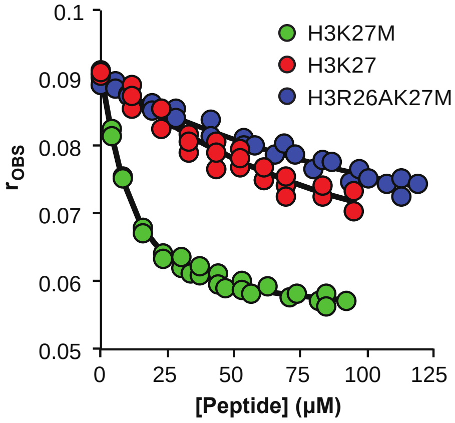

Peptide	Additive	Kd (μM)	rL	rPL
H3K27M	320 μM SAM	3.3 (0.4)	0.054 (0.002)	0.129 (0.002)
R26AK27M	320 μM SAM	73 (19)	0.054 (0.003)	0.127 (0.003)
H3K27	320 μM SAM	52 (12)	0.051 (0.002)	0.133 (0.001)

c. Titration with and without repressive peptide

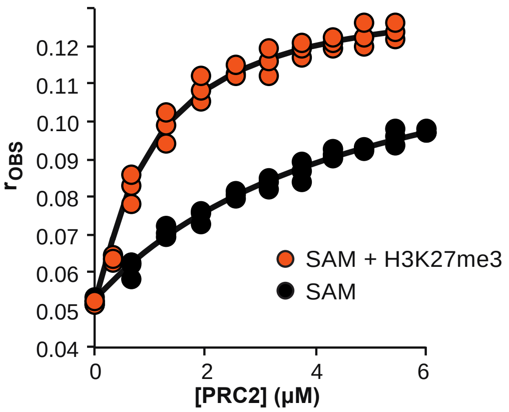

Peptide	Additive	Kd (μM)	rL	rPL
FAM-K27M	320 μM SAM<br>+H3K27me3	0.85 (0.21)	0.052 (0.001)	0.138 (0.002)

d. Titration with unlabelled peptide

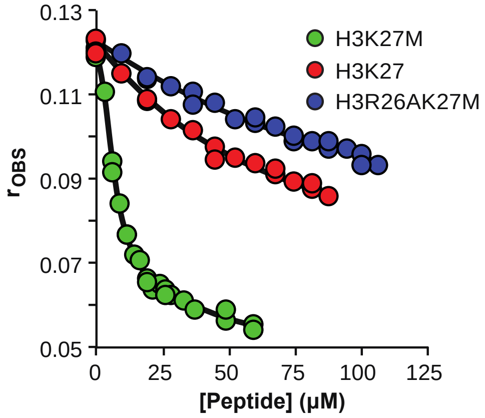

Peptide	Additive	Kd (μM)	rL	rPL
H3K27M	320 μM SAM<br>+H3K27me3	0.6(0.1)	0.050 (0.002)	0.137 (0.002)
R26AK27M		24 (6)	0.057 (0.004)	0.136 (0.003)
H3K27		13 (2.5)	0.053 (0.02)	0.133 (0.002)

d. Titration of SAM or SAH

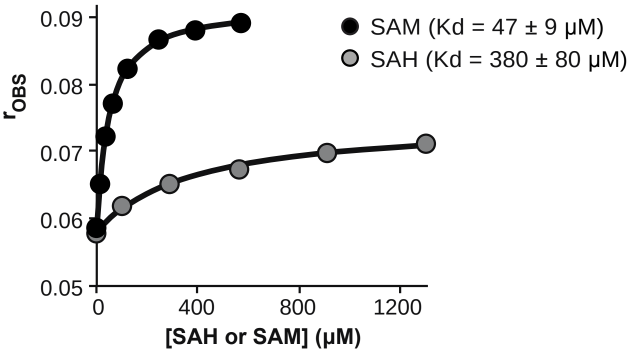

Supplementary Figure 4 – Peptide Binding to PRC2 Fluorescence spectroscopy, using a carboxy-fluorescein labelled H3K27M peptide (FAM-K27M), was used to measure the affinity of PRC2 for H3 peptide. A full description of the measurements and calculation is provided in Methods. (**a**) Direct titration of PRC2 into FAM-K27M (0.4 µM) in the presence of SAM (320 µM, black circles), in the presence of SAH (1.3 mM, grey circles), and in the absence of co-factor (white circles). Binding was stronger with SAM (Kd=4.3 µM) than SAH (Kd=14.6 µM) and in the absence of cofactor the binding of PRC2 was weak. (**b**) Displacement titrations of a mixture of PRC2 (4.32 µM), FAM-H3K27M (0.4 µM) and SAM (320 µM) with unlabelled peptides. (H3K27M (green circles), H3K27 (red circles), and H3R26AK27M (blue circles). The affinity of the unlabelled H3K27M is similar to that of FAM-H327M showing that the label does not interfere with the interaction. (**c**) Direct titration of PRC2 into FAM-K27M (0.4 □M) and SAM (320 µM) in the presence of repressive H3K27me3 peptide (orange circles). The titration in the absence of repressive peptide is reproduced from Supplementary Data Figure 4a for comparison (black circles). (**d**) Displacement titrations of a mixture of PRC2 (4.32 µM), FAM-H3K27M (0.4 µM), H3K27me3 (40 µM) and SAM (320 µM) with unlabelled peptides. (H3K27M (green circles), H3K27 (red circles), and H3R26AK27M (blue circles). (**e**) Titration of SAM or SAH into PRC2 (4.32 µM), FAM-H3K27M (0.4 µM). Analysis of these curves yields apparent binding constants of 47 ± 9 µM for SAM and 380 ± 80 µM for SAH.

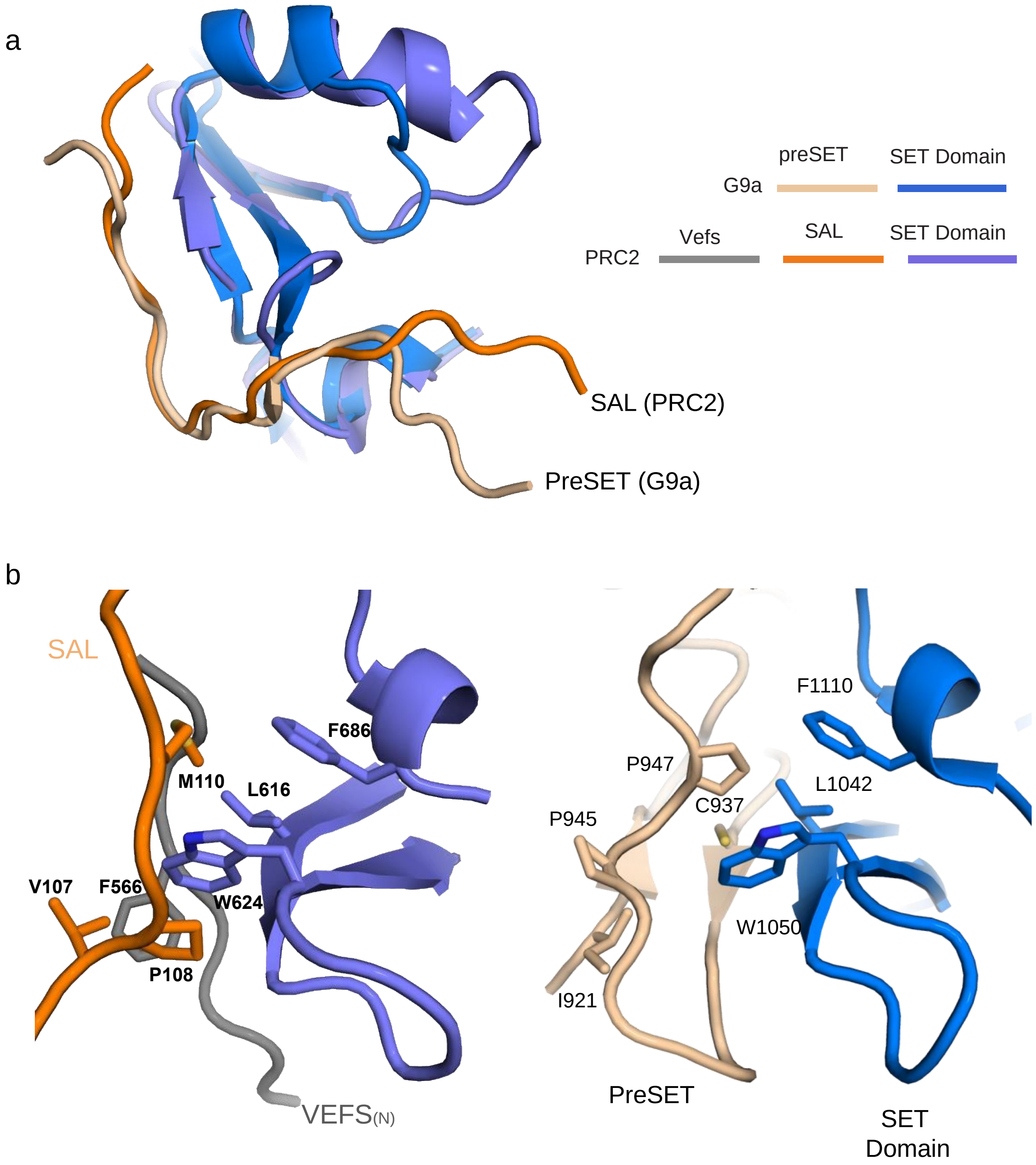

Supplementary Figure 5 – Comparison of SET domain stabilisation elements with G9a. Comparison of EZH2 with other SET domain structures reveals that different structural elements are used to provide equivalent stabilisation of the SET domain. The comparison with G9a (PDB: 2O8J) is particularly informative. (**a**) Superposition of the Set-I regions of PRC2 (blue) and G9a (beige) shows that the PRC2 SAL subdomain plays an analogous role to G9a residues 949-958 in stabilising SET-I. (**b**) Side by side comparison showing how the hydrophobic packing environment for the equivalent central residue of the Gly-x-Gly motif (Trp-624 in EZH2 and Trp-1050 in G9a) is provided by the SAL and VEFS N-terminal region in PRC2 (left) and by the PreSET domain residues Cys-937 to Pro-947 of G9a (right).

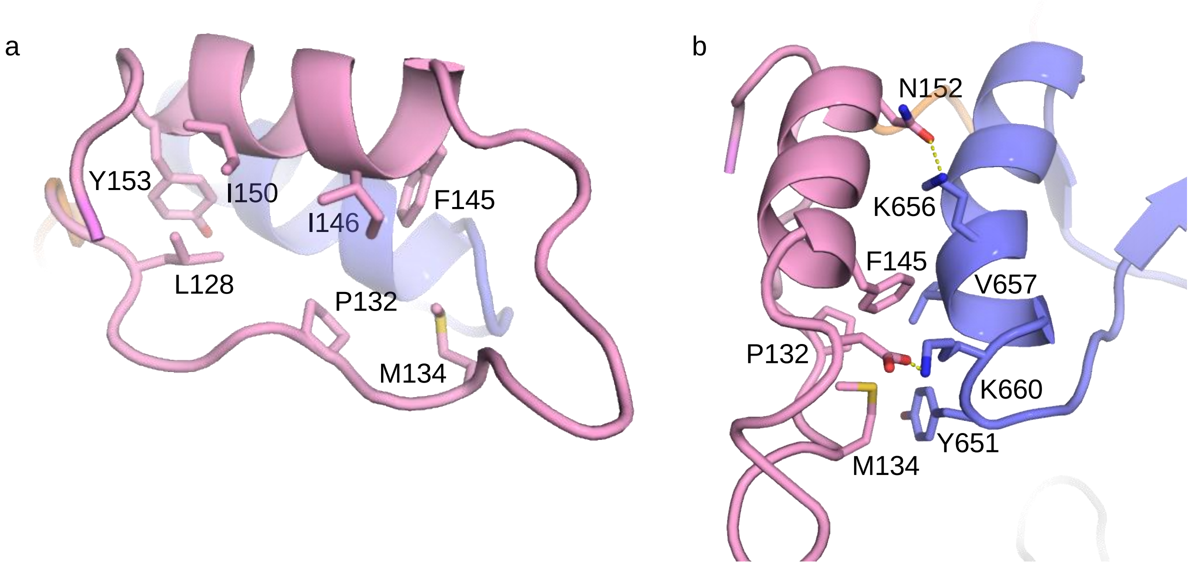

Supplementary Figure 6 – Binding of the SRM subdomain to EZH2. The repressive me3 peptide binds to EED through the SRM subdomain and stimulates EZH2 methylation. (**a**) Cartoon representation of the SRM subdomain (pink) and SET domain (blue) with key residues as sticks showing the arrangement of the hydrophobic side chains involved in interactions with SET-I on the SRM subdomain. (**b**) Interface between the SRM and SET-I helices.

	hsPRC2	ctPRC2 (stimulate)	ctPRC2 (Basal)
PDB code	5HYN	5CH1	5CH2
Normalised thermal factors $(\frac{Average\ B\ of\ SET-I\ helix}{Overall\ average\ B})^{*}$	0.729	0.903	1.547

*Average B factor was calculated by program BAVERAGE from CCP4 suite.

Supplementary Data Table 1: Temperature factors observed for the SET-I region in repressive SET domain structures. A feature of the activation of PRC2 methylation by binding of repressive marks to EED is stabilisation of the SET domain SET-I helix. To compare SET-I stabilisation we used an equivalent 9 residue segment from each SET-I helix (human PRC2 residues 648-657, yeast 833-842) and used the program BAVERAGE[33] to estimate the thermal factors for atoms in this segment.
